# Therapeutic Potential of Dexmedetomidine in Neuropsychiatric Disorders: From the Bench to the Clinic

**DOI:** 10.2174/011570159X349530241123140415

**Published:** 2025-01-02

**Authors:** Xiaojun Hu, Rong Luo, Fan Lei, Xin Li, Yuncheng Luo, Qingran Li, Limei Yi, Xia Zhang, Andrew Polyak, Yuanxiang Tao, Ruotian Jiang

**Affiliations:** 1 Department of Anesthesiology, West China Hospital, Sichuan University, Chengdu, 610041, China;; 2 Laboratory of Anesthesia and Critical Care Medicine, National-Local Joint Engineering Research Center of Translational Medicine of Anesthesiology, West China Hospital, Sichuan University, Chengdu, 610041, China;; 3 Department of Neurology, West China Hospital, Sichuan University, Chengdu, 610041, China;; 4 Rutgers Graduate School of Biomedical Sciences, Rutgers, The State University of New Jersey, New Brunswick, New Jersey, 07103, USA;; 5 Department of Anesthesiology, New Jersey Medical School, Rutgers, The State University of New Jersey, Newark, New Jersey, 07103, USA

**Keywords:** Dexmedetomidine, neuroprotection, anti-inflammatory, anti-apoptotic, neuropsychiatric disorders, α2 adrenergic receptor

## Abstract

Neuropsychiatric disorders encompass a range of conditions resulting from various dysfunctions within the nervous system, manifesting in diverse neurological impairments. These disorders, including depression, schizophrenia, anxiety, and Alzheimer's disease, impose significant economic and psychological burdens on both individuals and society overall. Recent clinical and preclinical studies have highlighted that dexmedetomidine (Dex), a highly selective α2 adrenergic receptor agonist, may offer therapeutic benefits beyond its well-known sedative properties. Dex has demonstrated neuroprotective effects, including anti-inflammatory and anti-apoptotic effects, as well as contributing to maintaining the integrity of the blood-brain barrier. Additionally, clinical observations suggest that Dex could be beneficial in managing neuropsychiatric disorders, with fewer side effects compared to traditional antipsychotics in both rodent and human studies. This review presents a comprehensive overview of the preclinical and clinical evidence supporting the therapeutic efficacy of Dex in neuropsychiatric disorders. We also discuss the underlying mechanisms of its effect and point out future research directions to further investigate Dex’s role in this field.

## 1 INTRODUCTION

Neuropsychiatric disorders encompass a wide spectrum of conditions that involve both neurological and psychiatric aspects, such as Alzheimer's Disease (AD), Parkinson's Disease (PD), depressive disorders, anxiety disorders, schizophrenia, bipolar disorder, autism spectrum disorders (ASD), epilepsy, and *etc*. [1]. Neuropsychiatric disorders continue to impose a substantial burden on society, consistently ranking among the top ten leading causes of disability since 1990 [2]. Its impact extends beyond individuals, affecting families and communities, thereby representing a significant public health problem globally. However, the molecular and cellular mechanisms underlying neuropsychiatric disease remain unclear, consequently causing a lack of highly efficient and specific therapeutic management. Currently, the treatment options for neuropsychiatric disorders may have limited efficacy for specific patients, making it difficult to fully alleviate symptoms, and they often come with significant side effects. Therefore, there is an urgent need to develop novel therapeutic drugs.

Dexmedetomidine (Dex), a sedative and hypnotic medication, was initially approved by the United States FDA in 1999 as a short-acting sedative (< 24 hours) for use in the Intensive Care Unit (ICU) [3]. By 2008, its use was extended to long-term sedation (over 24 hours) in the ICU, including for patients without tracheal intubation and those undergoing perioperative care [4]. Dex’s sedative effect is concentration-dependent: plasma levels between 0.2 and 0.3 ng/mL result in light sedation, allowing the patient to be easily roused, while deeper, unarousable sedation occurs at concentrations above 1.9 ng/mL [5, 6]. The sedative effect of Dex very resembles natural sleep, enabling the Dex-treated patients to exhibit greater states of alertness and cooperation, as compared to those taking other sedatives, such as propofol and midazolam [7-10]. Furthermore, Dex possesses anti-inflammatory, anti-sympathetic, analgesic, and other properties, which are likely associated with the reduction in the incidence of postoperative delirium [11]. These characteristics make Dex quickly integrated into widespread use as a sedative.

Although Dex was first used for its sedative effects, its diverse pharmacological actions suggested that it could also be effective in neuropsychiatric disorders. In 1993, Hoffman *et al*. first reported the neuroprotective effects of Dex, demonstrating in a rat model of cerebral ischemia that pre-treatment with Dex reduced intracranial catecholamine levels and mitigated neuronal damage [12]. This pioneer work promoted numerous researchers to investigate the neuroprotective properties of Dex. Preclinical studies have shown that Dex exerted its neuroprotective effects by inhibiting the release of inflammatory factors, suppressing apoptotic pathways, reducing the production of pro-apoptotic proteins, and safeguarding the integrity of the blood-brain barrier in some models of nerve damage [13-17]. In clinical settings, Dex has often been used in neurosurgery, since it effectively reduces brain metabolism, protects neurons, and does not affect neurophysiological monitoring [18]. It has been proven that the proper use of Dex can significantly relieve perioperative anxiety [19, 20]. In 2022, sublingual Dex (BXCL501) received approval for the treatment of agitation associated with schizophrenia and bipolar disorder in adult patients [21]. The availability of BXCL501 has expanded the clinical application of Dex, making it a valuable therapeutic option for neuropsychiatric disorders. Dex's neuroprotective effects hold promise for slowing the progression of these conditions.

This article attempts to provide a comprehensive overview of the mechanisms underlying Dex’s neuroprotective effects, with a particular emphasis on its application in neuropsychiatric disorders. Furthermore, we discuss the limitations of current research and propose directions for future investigations.

## 2 THE MOLECULAR AND CELLULAR MECHANISM OF ACTION OF DEX IN THE NERVOUS SYSTEM

The α2 adrenergic receptors (α2ARs) are subtypes of the G_i/o_ -protein-coupled receptor family. They can be selectively activated by Dex and are considered important molecular targets for Dex to exert its effect [22]. These α2ARs are widely distributed in the central nervous system, autonomic ganglia, and various peripheral organ tissues, including blood vessels, liver, kidney, pancreas, *etc*. [23].

The α2ARs exist in three subtypes: α2A, α2B, and α2C [22]. Among the three subtypes, α2A adrenergic receptors (α2A-ARs) have been extensively studied and are recognized as major presynaptic inhibitory feedback receptors, that regulate the release of norepinephrine (NE) [24]. Dex, as a highly selective agonist, can exert sedative and analgesic functions by activating α2A-ARs to control the release of NE [24, 25]. The α2B adrenergic receptors (α2B-ARs) are predominantly located in peripheral vascular smooth muscle and play an important role in regulating cardiovascular function [26, 27]. Activation of peripheral α2B-ARs causes sodium retention and peripheral vasoconstriction, indicating that α2B-ARs are involved in the maintenance of hypertension [27, 28]. The α2C adrenergic receptors (α2C-ARs) are mainly found in the hippocampus, basal ganglia, olfactory bulb system, and cerebral cortex [29]. Apart from modulating the release of NE, α2C-ARs also play a crucial role in regulating the release of dopamine, serotonin, and other neurotransmitters, which hold a significant influence on neuropsychiatric disorders [30]. The involvement of α2C-ARs is implicated in the regulation of intricate memory processes and the pathogenesis of neuropsychiatric conditions, including depressive disorder, AD, substance use disorder, and schizophrenia [29, 31, 32]. Pharmacological selective inhibition or transgenic knockout of α2C-AR has demonstrated anxiety alleviation and potential therapeutic benefits for cognitive deficits associated with schizophrenia, depressive disorder, and other related disorders [32-34]. These α2C-ARs’s functions indicate that α2C-AR may be a potential target for Dex in the treatment of neuropsychiatric disorders. It should be noted that these three receptor subtypes do not function independently but rather interact in conjunction under various physiological and pathological contexts.

While α2ARs are predominantly expressed in neurons, recent research indicates their presence in other cell types, including astrocytes and microglia. Studies using immunofluorescence and reverse transcriptase polymerase chain reaction (RT-PCR) confirmed α2A-AR expression on astrocytes, both *in vitro* and *in situ* in mouse brains [35-37]. However, there is still a controversial opinion among researchers regarding the presence of α2A-AR on microglia. An *in vitro* study demonstrated the expression of mRNA encoding the α2A-AR in lipopolysaccharide-activated cultured microglia through PCR assay [38]. In contrast, immunofluorescence staining of brain sections from a mouse sepsis model revealed the absence of α2A-AR in microglia located in the hippocampus [37, 39]. The different findings among these studies may be attributed to several factors. Firstly, *in vivo* and *in vitro* experiments may yield different results. Secondly, the differences in the experimental models carried out may influence the expression of genes involved in microglial function [39]. Future investigations can employ additional research techniques, such as RNA scope, cell-specific PCR, and WB to further elucidate the expression of α2A-AR in microglia under distinct experimental conditions.

The possibility that Dex may exert its effects by binding to α2ARs on glial cells, in addition to neurons, is an emerging area of interest. Previous investigations have emphasized the involvement of glial cells in the neuroprotective effects of Dex. In animal models of neuropsychiatric disorders, such as AD and anxiety, Dex demonstrated effective inhibition of microglial overactivation, prevention of astrocyte pyroptosis, and promotion of M2 microglia polarization, leading to the reductions in pro-inflammatory factors and axon demyelination [13, 40]. Consequently, these effects contribute to the amelioration of tissue damage, neuronal loss, and improvement in neurological function. However, whether Dex directly acts on α2ARs in glial cells remains unclear. Gao *et al.* reported that the anti-inflammatory effects of Dex on LPS-treated microglia could be blocked by α2ARs inhibitors, such as yohimbine, suggesting that Dex may interact with α2ARs in glial cells [41]. Nonetheless, further research is required to validate this conclusion. Conditional knockout of α2ARs in glial cells could provide insight into the mechanisms of Dex's action. Additionally, glial cells may participate in other effects of Dex, such as inhibiting the transitional activation of astrocytes and microglia under arthritis pain conditions and suppressing gap junction communication between astrocytes and neurons under general anesthesia [42, 43]. Determining whether glial cells are essential for Dex’s effects is crucial for identifying new therapeutic targets and developing novel drugs. The main proposed mechanisms of Dex functions are described in Fig. (**1**).

## 3 ROLE OF DEX IN NEUROPSYCHIATRIC DISORDERS

The neuroprotective effects of Dex have spurred a surge of interest in its potential to treat neuropsychiatric disorders. The evidence described below focuses on the mechanisms of Dex action and its clinical use in neuropsychiatric disorders. Tables **1** and **2** also summarize preclinical and clinical findings across multiple neuropsychiatric disorders.

### 3.1 AD

In individuals with AD, there are three key neuropathological features: β-amyloid (Aβ) deposition, neurofibrillary tangles containing hyperphosphorylated tau (P-tau), and neuroinflammation [44-47]. These characteristics are interconnected and mutually reinforce each other, leading to neurotoxicity and apoptosis [48-50].

Recent preclinical studies have highlighted the potential function of Dex in slowing the progression of AD. Dex effectively inhibited neuroinflammation and prevented neuron apoptosis under AD conditions [51-53] and could also alleviate cognitive deficits, a prominent symptom of AD, in mouse models of AD, as assessed by Morris water maze and Y maze tests [13, 15, 54]. Therefore, growing research is focused on uncovering the neuroprotective mechanisms of Dex in AD.

With regards to Aβ, Luo *et al*. discovered that Dex inhibited Aβ deposition, while Gou *et al*. demonstrated that Dex effectively suppressed Aβ-induced apoptosis in neuronal cells [13, 55]. These effects were inhibited by α2ARs antagonist, yohimbine, indicating that Dex may treat AD by acting on the α2ARs [13]. However, yohimbine has several limitations, including low efficiency and lack of selectivity. More importantly, it is unknown which specific a2AR subtype such as the a2A-AR, a2B-AR or a2C-AR subtype is involved in the treatment of Dex for AD.

Microglial activation is considered to play a significant role in the AD-related neuroninflammation [46]. Dex has been found to promote phenotypic transformation of microglia from an inflammatory activated state to a no nerve-damaging function both *in vitro* and *in vivo* works by increasing the expression of triggering receptors expressed on myeloid cells 2 (TREM2). The neuroprotective role of TREM2 has been confirmed through genetic investigations in AD [56-57]. Additionally, astrocyte activation contributes to AD pathology, particularly in Aβ deposition, though it is still unclear whether Dex directly affects astrocytes [58].

MicroRNAs (miRNAs), which regulate post-transcriptional gene expression, are key players in AD. They help modulate Aβ clearance by targeting multiple mRNA molecules, making them promising therapeutic targets [15, 59]. Some studies suggest that Dex can influence miRNA activity to maintain Aβ balance, promoting its clearance while also exhibiting anti-inflammatory, anti-apoptotic, and antioxidant effects [15, 55, 60]. Exploring the potential function of Dex in targeting miRNAs could provide another promising avenue for AD treatment.

However, controversial results have been reported regarding the effects of Dex on tau phosphorylation. Tau is abnormally hyperphosphorylated and forms neurofibrillary tangles in AD, resulting in neuronal degeneration and memory disorders [47]. A study revealed that Dex increased tau phosphorylation and impaired spatial memory [61]. These conflicting findings may be attributed to variations in the dosage and duration of Dex administration. Furthermore, the increase in tau phosphorylation caused by Dex appears to be a transient change.

Currently, an FDA-approved sublingual film formulation of Dex (BXCL501) has entered phase III clinical trials for the treatment of agitation associated with AD [62]. Nevertheless, it remains a paucity of clinical trials investigating the use of Dex in treating cognitive and sleep disorders, and other aspects of AD. A randomized, double-blind, placebo-controlled follow-up study is necessary for the assessment of the therapeutic effects of Dex on AD.

Overall, these studies reveal a potentially important role for Dex in the treatment of AD by regulating Aβ balance and anti-inflammation, implying that Dex may be a promising potential drug to alleviate AD progression and agitation.

### 3.2 PD

PD is a prevalent neurodegenerative disorder marked by the degeneration of dopaminergic neurons in the Substantia Nigra (SN). It is accompanied by various motor symptoms, including dyskinesia, muscle stiffness, resting tremors, and a spectrum of non-motor symptoms [63].

The *in vitro* experimental studies demonstrated that Dex reduced the production of Reactive Oxygen Species (ROS), regulated the cell cycle, and consequently attenuated apoptosis in all-trans-retinoicacid differentiated SH-SY5Y cells, treated with 1-methyl-4-phenylpyridinium (MPP^+^) for PD research [14]. However, it should be noted that *in vitro* experiments cannot fully replicate the complexities of the *in vivo* environment. Recent *in vivo* studies using mouse models of PD induced by methyl-4-phenyl-1,2,3,6-tetrahydropyridine (MPTP) have demonstrated that Dex reduces the degeneration of dopaminergic neurons, suppresses inflammation, and downregulates apoptotic proteins after a 3-7 day treatment [64, 65]. Furthermore, Zhou *et al*. reported that the neuronal mechanism by which Dex potentially modifies PD. They found that Dex decreased abnormal excitation in striatal neurons by reducing the excessive synaptic activity [64]. Notably, this effect was reversed by administrating a D2 antagonist, suggesting that Dex may exert its effects by influencing the D2 receptors. However, whether Dex directly acts on the D2 receptor requires further verification [64, 65]. Prior studies have examined the impact of Dex on dopamine: Dex diminishes extracellular dopamine levels in the nucleus accumbens and modulates histone H3 acetylation *via* the ERK1/2 signaling pathway in striatal dopamine neurons [66, 67]. These mechanisms likely contribute to Dex's neuroprotective properties, highlighting further investigations into the role of Dex within the dopamine system.

In clinical practice, Dex has extensive applications as a sedative in PD patients undergoing surgical procedures. In the context of PD, the management of general anesthesia presents challenges due to the impaired autonomic nervous system regulation function. These patients experience an increased cardiac burden following general anesthesia, which may potentially cause arrhythmias. Consequently, the utilization of local anesthesia and nerve blocks are often necessary to address these complexities. However, unstable autonomic activity poses a significant challenge in local anesthesia procedures. Recent case reports have shown that intraoperative sedation with Dex reduces the risk of autonomic instability, creating safer conditions for surgery [68-70]. Nevertheless, a future randomized, placebo-controlled study is necessary to determine the efficacy of Dex administration in patients with PD-related dyskinesia.

Dex also has extensive application in Deep Brain Stimulation (DBS), which is the primary treatment for advanced PD [71]. This surgical procedure involves implanting electrodes in specific brain regions to modulate abnormal neuronal firing patterns and alleviate motor and non-motor symptoms [71, 72]. To optimize electrode placement, physicians often use microelectrode recordings (MER) during surgery, which is typically performed with the patient awake to avoid interference from anesthetics [73, 74]. A recent study by Simon *et al.* demonstrated that Dex did not alter the characteristic subthalamic activity during DBS and provide good intraoperative sedation for patients. Another notable advantage of Dex was its ability to rapidly restore baseline subthalamic activity patterns in less than 10 minutes, even at a high dose of Dex [75]. However, it is crucial for the anesthesiologic team to diligently monitor the conscious state of the patient during this process. Furthermore, the patients who received Dex during DBS exhibited stable vital signs and did not experience significant side effects [76, 77]. These findings highlight the significant potential of Dex in PD surgeries.

In recent years, there has been a growing research focus on the clinical therapeutic potential of Dex for PD. A noteworthy case report in 2020 highlighted the efficacy of Dex in treating acute delirium in PD patients [78]. A prospective clinical study is to investigate the therapeutic effects of Dex in elderly patients with PD [79]. However, despite these advancements, there is still a need for further research to comprehensively understand the broader therapeutic benefits of Dex in a clinical context for PD.

In conclusion, current data highlight the important role of Dex in both the therapeutic management and surgical treatment of PD. Its neuroprotective effects and potential to alleviate symptoms suggest that Dex may be a valuable therapeutic agent for PD.

### 3.3 Epilepsy

Epilepsy, a chronic brain disorder characterized by abnormal and recurrent neuronal activity, poses a significant health concern, leading to premature mortality [80]. Current research explores both treatments for epilepsy and its underlying mechanisms. Studies in rat models have shown that Dex effectively reduces seizure severity, prolongs the onset of seizures, and decreases spike frequency in pentylenetetrazole-induced seizures. In a kainic acid-induced epilepsy model, Dex also demonstrated neuroprotective effects by reducing glutamate levels, preventing hippocampal neuron apoptosis, and upregulating neuroprotective proteins such as Bcl-2, Nrf2, and brain-derived neurotrophic factor [81-84].

In comparison to clonidine, which delays amygdala kindling but is less effective at controlling seizures and can even induce proconvulsive effects at higher doses, Dex offers a dual benefit of seizure control and neuroprotection [81, 84, 85]. Dex also shows potential for early detection of latent epilepsy, as seen in the Genetic Absence Epilepsy Rat from Strasbourg (GAERS) model of absence epilepsy, where it induced spike-and-wave discharges, a key marker of idiopathic generalized epilepsies-an effect not observed in wild-type mice [86, 87]. This report holds great clinical significance, as it expands the application of Dex in the clinical diagnosis and treatment of epilepsy.

In the context of epilepsy mechanisms, the dysregulation of NE is thought to play a critical role in epilepsy, with NE generally considered protective against seizures [88]. The α2ARs, as the receptors of NE, have also garnered significant attention regarding their role in epilepsy. The activation of the α2ARs receptor has been observed to inhibit epileptiform activity in the rat hippocampal CA3 region, an area associated with a high incidence of epilepsy [89]. Systemic administration of α2ARs antagonists, such as idazoxan, yohimbine, or rauwolscine, has been shown to accelerate amygdala kindling. Mice carrying a point mutation in the LC of α2ARs (the D79N mutant mice) exhibited a substantial loss of α2ARs function and were notably prone to amygdala kindling [88]. However, the existing evidence on the antiepileptic effects of Dex suffers from a lack of cell-specific α2ARs genetic tools and specific antagonist-related works. Consequently, a thorough investigation into the potential involvement of α2ARs in Dex-mediated effects is still warranted. Future research directions should consider knocking out α2ARs in specific brain regions and specific subtypes of α2ARs to address these gaps. There is also a paradox in Dex's antiepileptic action, as it is generally thought to inhibit NE release, yet still exerts anticonvulsant effects. This may be due to the low doses used in epilepsy studies (≤ 100 µg/kg), which may not significantly affect the LC, the brain's primary source of NE. In other words, Dex's antiepileptic efficacy might not stem from NE inhibition [9].

Clinically, Dex is widely used for sedation in epilepsy patients during examinations and surgery. It has proven particularly useful during magnetoencephalography (MEG) for presurgical evaluation, as it provides effective sedation with minimal impact on MEG spike frequency [90, 91].

In addition to accurately identifying the epileptogenic focus preoperatively, it is also essential to locate and map the seizure focus during surgery. Intraoperative electrocorticography (ECoG) is commonly employed for this purpose. However, the inhalation anesthetics used as intraoperative agents can significantly suppress brain electrical activity, thus affecting ECoG readings [92]. Dex either enhanced or did not alter the spike rate during intraoperative ECoG recording in most epilepsy surgeries. This suggests that Dex may hold potential utility in epilepsy surgery [92].

### 3.4 Anxiety

Some studies have indicated that Dex effectively reduces anxiety-like behaviors in animal models. Jang *et al*. provided valuable insights into this concern. They established rat models of Post-Traumatic Stress Disorder (PTSD) and observed that the administration of Dex expedited the extinction of fear memories and reduced anxiety levels [93]. These findings suggest that Dex possesses an anti-anxiety effect independent of its sedative, analgesic, and anti-inflammatory properties.

Dex has demonstrated its efficacy in alleviating anxiety in socially frustrated mice, through the inhibition of corticotropin-releasing hormone-producing hypothalamic paraventricular neurons (CRH^PVH^). Manipulating the activation or inhibition of CRH^PVH^ through chemogenetic assay was shown to modulate the anti-anxiety effects of Dex [94]. These findings underscore the significance of CRH^PVH^ as a crucial target for the anti-anxiety actions of Dex. Moreover, the inhibitory effect of Dex on CRH^PVH^ may be mediated through α2ARs [94]. This study represents a significant advancement in understanding the mechanism underlying the anti-anxiety effects of Dex, as it identified the specific nuclei and neurons involved for the first time. However, systemic administration of yohimbine in these studies may not fully inhibit α2ARs, and the specific receptor subtype and brain regions involved are still unclear. Future research should explore Dex’s anxiolytic mechanism using brain-region- and cell-type-specific α2AR knockdown techniques.

Clinically, Dex has gained popularity for managing anxiety during the perioperative period, with both intravenous and intranasal administrations showing effectiveness, especially in children [19, 20, 40, 95, 96]. Furthermore, when compared to other drugs (such as fentanyl and midazolam) commonly used during the perioperative period, Dex appears to exhibit superior anti-anxiety effects [95, 97-99]. However, the available evidence remains limited. Larger randomized controlled trials are necessary to establish the role of Dex as a treatment for anxiety. Additionally, current research primarily focuses on the effects of Dex in the perioperative period. Its efficacy in treating neurotic anxiety has yet to be conclusively proven.

### 3.5 Depressive Disorder

Major Depressive Disorder (MDD) is one of the most prevalent psychiatric disorders globally and stands as a leading cause of disability [100]. It affects approximately 7% of the population in the United States [101, 102].

Numerous studies have consistently demonstrated that depressive disorder has been associated with elevated levels of NE [103, 104]. Dex, known for its action on the α2A-ARs in the LC, acts by inhibiting the release of NE [101]. This suggests that Dex may be a potentially effective treatment for depression. In 2018, Moon *et al*. first conducted a study in which they found that Dex could alleviate depression in a rat model induced by sleep deprivation [105]. By the use of the assessments, such as the forced swimming test and tail suspension test, an increase in immobility latency was observed in the sleep-deprived model, indicating depression. In addition, the analyses of brain tissue using WB and ELISA assays revealed decreases in dopamine receptors and tryptophan hydroxylase, along with an increase in tyrosine hydroxylase, suggesting an abnormal increase in dopamine release. The administration of low-dose intraperitoneal Dex significantly abated depression-like behavior and normalized dopamine release in sleep-deprived mice [105]. This study not only provided strong evidence for the antidepressant effects of Dex but also unveiled its potential influence on the dopaminergic system. Moreover, other depression models have also corroborated the antidepressant effects of Dex, demonstrating its ability to alleviate depressive symptoms induced by chronic constrictive injury through the promotion of neurogenesis in the hippocampus [106].

In addition, Dex possesses several advantages over other antidepressant medications. Unlike ketamine, Dex demonstrated a lesser impact on cognitive abilities, memory, and emotional function, primarily due to its protective effects on limbic regions [107]. Moreover, previous studies revealed that Dex could mitigate the learning and memory deficits induced by ECT in depressed rats [108]. These advantageous characteristics of Dex form the foundation for its potential clinical approval as an antidepressant.

In clinical practice, Dex is commonly used as an adjunctive medication in conjunction with ECT. Depressed patients who received Dex alongside ECT showed improved antidepressant effects, reduced agitation, and more stable vital signs [109]. Furthermore, clinical trials have indicated that postpartum administration of Dex could significantly decrease the incidence of postpartum depression. However, there is a scarcity of clinical trials specifically investigating the therapeutic effects of Dex in patients with depression [110, 111].

Recently, a new study suggested that Dex might emerge as a potent alternative to ECT as an antidepressant treatment [112]. In this study, patients with MDD were randomly assigned to receive either Dex infusions or ECT treatment. The Dex infusion or ECT treatment was administered once every three days for a total of 10 sessions. The results revealed that Dex exhibited rapid and long-lasting antidepressant properties with fewer side effects compared to ECT [112].

These findings indicate that Dex holds promise as an antidepressant drug; however, further clinical trials are necessary to validate these results. Furthermore, previous investigations into the antidepressant properties of Dex predominantly employed intravenous administration. However, treating depression typically requires a prolonged course of therapy. Intravenous administration is an invasive route, posing challenges for the clinical use of Dex as an antidepressant. Notably, Dex has recently become available in sublingual form (BXCL501) and has achieved approval for the management of agitation. Future studies should focus on the effectiveness of this sublingual administration on depression and compare its efficacy to intravenous administration. Additionally, the absence of specific α2ARs inhibitors and gene tools for investigating the antidepressant properties of Dex presents a limitation in current research. There remains uncertainty regarding the precise target of the antidepressant effects of Dex. Further exploring the mechanism of Dex’s action would be valuable for advancing the development of novel antidepressant medications.

### 3.6 Agitation Associated with Schizophrenia and Bipolar Disorder

Acute agitation commonly observed in emergency departments or inpatient settings is often associated with psychosis. It can escalate into aggressive behavior if acute agitation is untreated. Previous studies have predominantly focused on the preventive and alleviating effects of Dex in postoperative agitation [113]. However, recent studies have also demonstrated the therapeutic efficacy of Dex in managing agitation resulting more specifically from mental illness. The treatment options for acute agitation and aggression in patients with severe mental illness primarily rely on benzodiazepines and first- and second-generation antipsychotics [114]. Nevertheless, the significant side effects and invasive administration route of these drugs fall short of meeting current clinical requirements. Recent studies have shown that, in individuals with mild to moderate agitation related to bipolar disorder, the administration of 120 μg or 180 μg of BXCL501 significantly reduced agitation scores at the 2-hour mark compared to control participants [115].

BXCL501 offers several distinct advantages over commonly used antipsychotic medications and benzodiazepines for managing agitation. Firstly, Dex is associated with a lower incidence of side effects such as nausea, vomiting, insomnia, and extrapyramidal symptoms compared to drugs like haloperidol [116]. Secondly, it poses a lower risk of delirium while keeping patients easily rousable [7, 117]. Additionally, sublingual administration provides a non-invasive and gentle approach, promoting patient cooperation during treatment [118].

The limitations exist regarding the study of Dex in the treatment of agitation. Firstly, most clinical trials have only examined its effects after a single episode. It remains unclear whether Dex can fundamentally reduce the frequency of manic episodes in patients with bipolar disorder or schizophrenia. Trials have also focused on mild to moderate agitation, raising questions about its efficacy in more severe cases. Moreover, it is also important to acknowledge the presence of mild side effects, including a slower onset compared to intramuscular injections, as well as somnolence, thirst, and hypotension, though no serious complications have been reported [115, 119].

Despite the clinical approval of Dex for treating agitation, the underlying mechanism of its action remains unclear due to a lack of experimental research investigating the downstream targets through which Dex exerts its anti-agitation effects. In addition, one major challenge impeding relevant basic research is the absence of precise behavioral detection methods for assessing agitation in animal models. Traditional approaches relying on static parameters are insufficient for meaningful detection in animals displaying agitation or accurately identifying its subtypes.

To address these limitations, Cao *et al*. recently introduced a machine-learning-based method to overcome these challenges [120]. Using postoperative delirium (POD) schema, they proposed a comprehensive approach involving pose evaluation, behavior analysis, and action sequence evaluation to differentiate between the non-POD group and the POD group [120]. By employing a multi-scaled clustering analysis framework, these researchers successfully captured the hierarchical dynamics of delirium-like behaviors [120]. Notably, their findings showed that Dex reduced both the severity and incidence of POD in the animal model [120].

This innovative method not only enhances the accuracy of model construction but also enables the distinction of agitation subtypes. In the future, the researchers can explore the application of this approach to establish animal models of agitation and other neuropsychiatric diseases, facilitating investigations into the pathogenesis of these conditions and the therapeutic mechanisms of Dex. Such endeavors hold significant implications for understanding neuropsychiatric diseases and developing promising therapeutic interventions.

### 3.7 ASD

ASD is a neurodevelopmental disorder characterized by social difficulties, presenting with behavior commonly stereotyped for the inability to detect social cues [121]. In the United States, the prevalence of ASD has been steadily increasing, with approximately 2.3% of children and 2.2% of adults affected by the condition. The majority of individuals with ASD require assistance in daily living, placing a significant burden on both society and families.

In previous studies, Dex has demonstrated notable neuroprotective effects and exhibited positive therapeutic outcomes in cognitive and mood disorders [13]. These characteristics enable the researchers to turn their attention to investigating the potential therapeutic effects of Dex in ASD. Liang *et al.* conducted pioneering research and revealed the therapeutic effects of Dex on social disorders and stereotypical behavior in BTBR T^+^ Itpr3^tf^ /J (BTBR) mice, an established ASD model through three-chamber, self-grooming, marble burying, open field, and elevated plus maze [122]. Furthermore, this study uncovered the underlying mechanisms and identified that Dex exerted its effects by inhibiting the release of the inflammatory cytokine IL-6 and modulating abnormal excitation of glutaminergic neurons in the prelimbic cortex through α2ARs activation [122]. These findings provide initial evidence supporting the beneficial effects of Dex on the core symptoms of ASD, thereby offering a theoretical foundation and potential therapeutic strategy for clinical interventions in ASD. Future studies could delve deeper into the involvement of other brain regions, encompassing both neurons and glial cells, to further elucidate the mechanisms underlying the therapeutic effects of Dex in ASD.

In clinical practice, Dex is primarily used as a sedative to facilitate necessary tests for individuals with ASD. Intranasal administration of Dex has demonstrated effective sedation and anxiety reduction in ASD patients [123-125]. However, intranasal administration may potentially cause psychological distress and increase the resistance to medical examinations among ASD patients. The introduction of BXCL501 presents a potential improvement in this regard. Future clinical investigations could explore the use of sublingual administration to achieve satisfactory sedation effects. Furthermore, given that there is currently a dearth of clinical studies examining the therapeutic potential of Dex in treating ASD, it is important for researchers to design appropriate clinical studies in this area to ascertain whether Dex indeed holds therapeutic efficacy for individuals with ASD.

## 4 PHARMACOLOGICAL PROPERTIES AND SAFETY OF DEX

Dex undergoes primary elimination *via* hepatic biotransformation [126]. It exhibits a rapid distribution half-life of approximately 6 minutes, characterized by swift onset, with an elimination half-life of around 2 hours [127]. The relatively long half-life necessitates close monitoring of vital signs during medication to mitigate risks like respiratory depression. Despite its current utility, safety concerns persist with the use of Dex, primarily due to the absence of a rapidly acting reversal agent and is mostly administered intravenously in clinic, thereby significantly restricting its clinical application as a therapeutic intervention in neuropsychiatric disorders. The side effects of Dex mainly focus on its impact on hemodynamics, encompassing transient hypertension, hypotension, and bradycardia, induced by mechanisms such as vasoconstriction, sympathetic inhibition, and baroreflex-mediated parasympathetic activation [5, 6]. These side effects in the treatment of neuropsychiatric disorders mirror those observed during the sedative administration of Dex [115]. While no severe adverse events have been reported, it remains essential to optimize the treatment regimen and diligently monitor patients' vital signs when administering Dex.

The management of neuropsychiatric disorders typically entails long treatment durations, often extending over several years or a lifetime. One limitation of current antipsychotics is the risk of developing resistance over time. Most research on Dex in neuropsychiatric disorders has focused on short-term use, but more studies are needed to assess the long-term viability and potential for resistance [52, 105]. Besides, the treatment of neuropsychiatric disorders commonly necessitates a blend of multiple medications to achieve optimal therapeutic outcomes. Nevertheless, intricate treatment regimens may heighten the occurrence of side effects in patients. Currently, there is limited research on the combination of Dex with antipsychotics. Existing studies have primarily explored its anesthetic use, where Dex, combined with agents like sevoflurane or propofol, reduces the dosage of anesthetics and minimizes related side effects [128, 129]. Some studies have indicated that the combination of Dex and ketamine yields superior results in alleviating preoperative anxiety in children compared to Dex alone, withoutadditional adverse effects [130, 131]. Future research should focus on evaluating the therapeutic efficacy and safety of Dex when used alongside traditional antipsychotics, which could enhance treatment strategies and support the broader clinical use of Dex for neuropsychiatric disorders.

## CONCLUSION AND FUTURE DIRECTIONS

In summary, Dex exhibits neuroprotective effects. In addition to its clinical use as a sedative and analgesic, Dex may hold promise as a potential option for the treatment of neuropsychiatric disorders. Despite the promising potential, much of the current research on Dex’s application in neuropsychiatric disorders is based on preclinical animal studies, and translating these findings into clinical practice remains a significant challenge. This may be attributed to several factors: (1) Animal models may not fully capture the intricacies of human neuropsychiatric disorders; (2) The underlying pathophysiology of many neuropsychiatric conditions is still poorly understood; (3) There are notable differences in higher brain network organization between rodents, even non-human primates, and humans, particularly in the types, localization, and density of specific neurons and receptor subtypes. These factors underscore the need for caution when extrapolating animal data to human clinical applications and highlight the importance of further research in this area. In addition, careful consideration should be given to factors such as dosage, frequency of administration, and treatment duration, as they can significantly influence outcomes. These considerations are crucial in study design and result interpretation to determine the optimal medication approach.

Certain questions regarding the mechanisms of Dex in these disorders remain unanswered. Specifically, the direct target of Dex in the treatment of neuropsychiatric diseases remains largely unknown. Further studies are required to elucidate how Dex targets specific brain regions and cell types in animal models of different neuropsychiatric disorders. A comprehensive understanding of these mechanisms will not only verify the Dex functions in these neuropsychiatric disorders but also facilitate the development of new drugs for these disorders.

Collectively, the reviewed evidence indicates that Dex could have therapeutic potential in various neuropsychiatric disorders. The identification of the neuroprotective properties of Dex, along with ongoing investigations into its mechanisms of action, offers promising insights into its possible applications in treating these conditions. These insights not only enhance our understanding of the broad application of Dex across diverse neuropsychiatric conditions but also refine our comprehension of the underlying pathophysiology. Importantly, they are anticipated to yield novel discoveries in the future. The FDA has approved the use of BXCL501 for agitation associated with schizophrenia and bipolar disorder in adult patients. Unlike traditional intravenous administration, sublingual administration offers distinct advantages, including enhanced patient cooperation and comfort, while also enabling the potential for long-term drug administration in individuals with neuropsychiatric conditions. The federal approval of BXCL501 may contributed to a broader clinical use of Dex as a therapeutic option for neuropsychiatric diseases.

## Figures and Tables

**Fig. (1):**
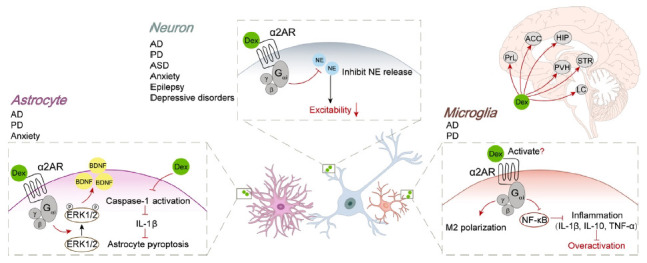
The mechanism of dex in treating neuropsychiatric disorders in the central nervous system. The therapeutic mechanism of Dex in addressing neuropsychiatric disorders encompasses various brain regions, such as the HIP, ACC, PrL, PVH, STR, and LC. Dex exerts an inhibitory effect on neuronal hyperactivity, thereby offering treatment benefits for several neuropsychiatric disorders, including AD, PD, ASD, anxiety, epilepsy, and depressive disorders. In glia, Dex demonstrates effective prevention of astrocyte pyroptosis, inhibition of microglial overactivation, and promotion of M2 microglia polarization, consequently diminishing the production of pro-inflammatory factors. **Abbreviations:** α2AR, α2 adrenergic receptor; Dex, dexmedetomidine; HIP, hippocampus; ACC, anterior cingulate cortex; PrL, prelimbic cortex; PVH, hypothalamic paraventricular; STR, striatum; LC, locus coeruleus; AD, Alzheimer’s disease; PD, Parkinson’s disease; ASD, autism spectrum disorders; BDNF, Brain-derived neurotrophic factor.

**Table 1:** Preclinical studies of Dex in neuropsychiatric diseases.

**Diseases**	**Study Model**	**Therapeutic Regimen (Dex)**	**Main Findings**	**References**
AD	AD mice model induced by Aβ injection or 5 x FAD mice	i.p. (20 or 100 μg/kg) for several days	Dex slowed the progression of AD and improve its cognitive deficits.	[51-55]
PD	PD mice model-induced by MPTP	i.p. (2~50 μg/kg) for several days	Dex inhibits inflammation and the degeneration of dopamine neurons.	[64-65]
Epilepsy	Epilepsy rats model induced by PTZ or GAERS rats.	i.p.; 100~500 μg/kg	Dex provided a dual benefit of seizure control and neuroprotection.	[81-87]
Anxiety	PTSD or SDS model	i.p.; 20 or 40 μg/kg	Dex effectively alleviated anxiety levels.	[93-94]
Depressive disorders	Sleep deprivation or CCI mice model	i.p. (0.5~25 μg/kg) for several days	Dex significantly abated depression-like behavior and normalized dopamine release.	[105-106]
ASD	BTBR mice	i.p. (0.8 μg) for several days	Dex treated social disorders and stereotyped behavior in BTBR mice.	[122]

**Abbreviations**: Aβ: β-amyloid MPTP: methyl-4-phenyl-1,2,3,6-tetrahydropyridine i.p.: intraperitoneal injection, PTSD: Post-Traumatic Stress Disorder SDS: social defeat stress CCI: chronic constrictive injury, Dex: dexmedetomidine AD: Alzheimer's disease PD: Parkinson's disease ASD: Autism spectrum disorder.

**Table 2:** Clinical studies of Dex in the neuropsychiatric diseases.

**Diseases**	**Subjects**	**Therapeutic Regimen (Dex)**	**Main Findings**	**References**
AD	AD patients	Sublingually; 30 mcg, 60 mcg or 90 mcg;	The administration of Dex demonstrated a significant reduction in the agitation score among patients with AD, with 60 mcg being the most effective dosage.	[62]
PD	PD patients	Continuous intravenous infusion at a dosage of 0.2~0.6 mcg kg^-1^ h^-1^	Dex decreased effectively involuntary movement during surgical procedures without interfering with nerve activity in DBS.	[68-70, 75-77]
Epilepsy	Epilepsy patients who are unable to cooperate	Intravenous or intranasal; 2 mcg kg^-1^	In the diagnosis and treatment of epilepsy, Dex exhibited effective sedation properties without impacting the electrical activity of the brain as observed in MEG and ECoG.	[90-91]
Anxiety	Adults and children with preoperative anxiety	Intravenous (0.2~0.5 mcg kg^-1^ or intranasal (1~3 mcg kg^-1^)	Dex reduced anxiety levels obviously both before and after surgery.	[19-20, 95-96]
Depressive disorders	Depressed patients and parturients	Intravenous (0.5 mcg kg^-1^) pre or post ECT	Dex augmented the antidepressant effects of ECT and demonstrated potential in preventing postpartum anxiety.	[109-111]
Agitation	Schizophrenia and bipolar disorder patients	Sublingual; 120 mcg or 180 mcg	The sublingual administration of Dex exhibited a significant reduction in 2-hour agitation scores.	[115]
ASD	ASD patients	Intravenous; 1~5 mcg kg^-1^	Dex demonstrated effective sedation to facilitate necessary tests for individuals with ASD.	[123-125]

**Abbreviations**: Dex: Dexmedetomidine AD: Alzheimer's disease PD: Parkinson's disease ASD: Autism spectrum disorder, ECT: Electroconvulsive therapy DBS: Deep brain stimulation MEG: Magnetoencephalography ECoG: Electrocorticography.

## LIST OF ABBREVIATIONS

AD: Alzheimer's Disease
ASD: Autism Spectrum Disorders
Aβ: β-amyloid
α2A-ARs: α2A Adrenergic Receptors
α2ARs: α2 Adrenergic Receptors
α2B-ARs: α2B Adrenergic Receptors
α2C-ARs: α2C Adrenergic Receptors
BTBR: BTBR T+ Itpr3tf/J
CRH^PVH^: Corticotropin-releasing Hormone-producing Hypothalamic Paraventricular Neurons
DBS: Deep Brain Stimulation
Dex: Dexmedetomidine
ECoG: Electrocorticography
GAERS: Genetic Absence Epilepsy Rat from Strasbourg
MDD: Major Depressive Disorder
MEG: Magnetoencephalography
MER: Microelectrode Recordings
miRNAs: MicroRNAs
MPTP: Methyl-4-phenyl-1,2,3,6-tetrahydropyridine
NE: Norepinephrine
P-tau: Hyperphosphorylated Tau
PD: Parkinson's Disease
POD: Postoperative Delirium
PTSD: Post-traumatic Stress Disorder
ROS: Reactive Oxygen Species
RT-PCR: Reverse Transcriptase Polymerase Chain Reaction
SN: Substantia Nigra
TREM2: Triggering Receptors Expressed on Myeloid Cells 2
